# Respiratory Infection-Related Pathogens in the Pediatric Intensive Care Unit During 2019–2024 in Hubei, China

**DOI:** 10.3390/pathogens15020219

**Published:** 2026-02-14

**Authors:** Jiahui Chen, Ying Li, Dan Sun, Hebin Chen, Haizhou Liu, Wenqing Li, Yanli Wang, Feng Han, Jiali Xu, Xueru Liu, Hui Du, Youjing Liu, Qing Du, Yifei Zhang, Yan Li, Yi Yan, Di Liu, Xiaoxia Lu

**Affiliations:** 1 Department of Respiratory Medicine, Wuhan Children’s Hospital, Tongji Medical College, Huazhong University of Science and Technology, Wuhan 430014, China; jiahuichen994@gmail.com (J.C.); elise_lii@163.com (Y.L.); abin319@126.com (H.C.); liwenqing@zgwhfe.com (W.L.); wangyanli5@aliyun.com (Y.W.); hanfeng@zgwhfe.com (F.H.); xujiali1@zgwhfe.com (J.X.); liuxueru@zgwhfe.com (X.L.); 13407180763@163.com (H.D.); liuyoujing05@126.com (Y.L.); zer1990@163.com (Q.D.); yanli@hust.edu.cn (Y.L.); 2 State Key Laboratory of Virology and Biosafety, Wuhan Institute of Virology, Chinese Academy of Sciences, Wuhan 430071, China; liuhz@wh.iov.cn; 3 School of Life Science and Health, Hunan University of Science and Technology, Xiangtan 411201, China; zhangyf@hnust.edu.cn; 4 Sanya Institute of Hunan University of Science and Technology, Sanya 572024, China; 5 Pediatric Respiratory Disease Laboratory, Institute of Maternal and Child Health, Wuhan Children’s Hospital, Tongji Medical College, Huazhong University of Science and Technology, Wuhan 430014, China; 6 University of Chinese Academy of Sciences, Beijing 101409, China; 7 Department of Critical Care Medicine, Wuhan Children’s Hospital, Tongji Medical College, Huazhong University of Science and Technology, Wuhan 430014, China; sundan@zgwhfe.com; 8 Department of Pathogen Biology, School of Basic Medicine, Tongji Medical College and State Key Laboratory for Diagnosis and Treatment of Severe Zoonotic Infectious Diseases, Tongji Medical College, Huazhong University of Science and Technology, 13 Hangkong Road, Wuhan 430030, China

**Keywords:** epidemiology, pediatric intensive care unit, pathogen spectrum, respiratory infections

## Abstract

Respiratory infections are a leading cause of hospitalization and mortality in children, and the pediatric intensive care unit (PICU) is a critical setting for managing severe cases. However, the epidemiological patterns of respiratory pathogens in the PICU remain insufficiently characterized. In this retrospective study, we analyzed respiratory pathogen testing results from 2126 pediatric patients admitted to the PICU of Wuhan Children’s Hospital between 2019 and 2024. The pathogen spectrum and epidemiological characteristics were evaluated across age groups and seasons. Respiratory syncytial virus (RSV, 18.06%) was the most frequently detected viral pathogen, while *Streptococcus pneumoniae* (6.96%) was the predominant non-viral pathogen. The overall infection burden was highest in children aged ≤ 1 year (53.75%) and 3 < age ≤ 6 years (54.70%), indicating that early childhood represents a high-risk period for severe respiratory infections requiring intensive care. Pathogen distribution varied significantly across age groups. Distinct seasonal patterns were observed for several respiratory pathogens, particularly among viral pathogens, whereas non-viral pathogens showed more variable seasonal distributions. Furthermore, screening for 10 common pathogens accounted for 75% of PICU respiratory infections, highlighting the clinical utility of multiplex molecular detection. This study delineates the pathogen spectrum of respiratory tract infections in the PICU and characterizes their age- and season-specific epidemiological patterns. This study defines the pathogen spectrum and age- and season-specific patterns of respiratory infections in the PICU, providing evidence to support targeted pathogen surveillance, optimized multiplex diagnostics, and risk-informed infection control strategies in pediatric critical care.

## 1. Introduction

Respiratory tract infections (RTIs) are the most common infectious diseases among children globally, with particularly heavy burdens in children under five years of age due to their high incidence and hospitalization rates [1,2]. According to the Global Burden of Disease Study published in The Lancet, RTIs are responsible for more than 1.5 million deaths annually in children under five years old, accounting for nearly 20% of total childhood mortality worldwide, making them one of the leading causes of death in this age group [3,4]. In China, RTIs are also a major cause of pediatric clinic visits and hospital admissions, seasonal respiratory infections are a leading cause of pediatric hospitalizations, especially in autumn and winter [5]. Although many RTIs are mild and self-limiting, they can rapidly progress to severe conditions such as pneumonia, acute respiratory failure, and even acute respiratory distress syndrome (ARDS), particularly in high-risk populations including infants, immunocompromised children, and those with underlying conditions [6]. These critically ill patients often require advanced life support in pediatric intensive care units (PICUs). RTIs are also among the most common infectious causes of PICU admissions, imposing a significant burden on critical care resources. Studies have shown that respiratory diseases account for 30–50% of total PICU admissions in both China and other Asian countries, with RTIs being the predominant etiology [7,8]. These critically ill children often require advanced support such as invasive or non-invasive mechanical ventilation, intravenous nutrition, and antimicrobial therapy, and typically experience longer PICU stays, higher severity indices, and elevated mortality compared to non-ICU patients. For instance, RSV infections in pediatric populations have been linked to prolonged ICU admission, requirement for positive-pressure ventilation, and increased mortality [9]. Additionally, long-stay PICU patients have been shown to consume significantly more intensive care resources and face a heightened risk of complication [10]. Therefore, identifying the pathogen spectrum and epidemiological patterns of RTIs in the PICU setting is essential for improving early diagnosis, optimizing anti-infective strategies, and enhancing the efficiency of diagnostic techniques.

RTIs in children are caused by a wide range of pathogens, including viruses, bacteria, mycoplasma, and fungi. Common viral pathogens include respiratory syncytial virus (RSV) [9,11], influenza virus (types A and B, FluA and FluB), human metapneumovirus (hMPV), adenovirus (ADV), human parainfluenza virus (HPIV), and rhinovirus (HRV) [12], while bacterial pathogens often involve *Streptococcus pneumoniae* (*S. pneumoniae*), *Staphylococcus aureus* (*S. aureus*), *Haemophilus influenzae* (*H. influenzae*), and *Mycoplasma pneumoniae* (MP) [12,13,14,15,16]. In recent years, fungal infections have also gained attention, particularly in immunocompromised children or those receving prolonged antibiotic treatment in PICUs [17]. The prevalence and seasonal patterns of these pathogens vary by geography, climate, and demographic factors [18,19]. Given the complex etiology and overlapping clinical symptoms, molecular diagnostics such as qPCR and multiplex PCR are indispensable for early and precise pathogen detection.

To date, most studies on the RTI pathogen spectrum in China have been based on large-scale surveillance of outpatients or general hospitalized populations rather than PICU cohorts. A nationwide multicenter investigation identified RSV, ADV, hMPV, and influenza virus as the leading pathogens in pediatric RTIs [20], while a separate national surveillance study (2009–2020) highlighted the frequent occurrence of viral–MP coinfections in community-acquired pneumonia, reflecting the substantial burden of mixed infections in children [21,22]. However, these studies primarily targeted mild-to-moderate infections and lacked in-depth analysis of critically ill patients. In the PICU context, children often present with complex comorbidities, rare pathogens, and multidrug-resistant organisms, necessitating more specialized surveillance. Although a few studies have reported on PICU infections [23,24], these were generally limited by sample size, time span, or detection breadth, and systematic investigations remain lacking. Although the present study expanded pathogen coverage using multiple diagnostic modalities, detection breadth may still be influenced by clinically driven testing practices, particularly for rare or emerging pathogens.

As a major metropolitan center and transportation hub in central China, Wuhan plays a pivotal role in the spread of respiratory pathogens due to its high population mobility and extensive interregional connections. Studies have shown that cities with high mobility and transportation centrality are at greater risk of acting as sources of pathogen spread, especially during winter epidemic seasons [25,26]. Several surveillance-based studies in Wuhan have characterized the temporal dynamics of respiratory pathogens among hospitalized pediatric patients. For example, an eight-year longitudinal surveillance study conducted in a major hospital in Hubei Province (2014–2022) reported a marked increase in RSV detection rates among hospitalized children since 2018, with a consistent winter seasonal peak pattern [27]. In addition, surveillance data from Wuhan between 2018 and 2021 showed that RSV remained the predominant respiratory virus among pediatric patients, accompanied by substantial circulation of ADV, hMPV, and IFV, highlighting the sustained burden of multiple respiratory viruses in this region [28]. And there are such studies have clarified the epidemiology characteristics of respiratory viruses spanning and after the COVID-19 pandemic among children with LRTIs [29,30]. Despite these efforts, no comprehensive research has yet been conducted to systematically examine the pathogen spectrum, epidemiological trends, or diagnostic performance associated with RTIs in children admitted to PICUs in Wuhan, leaving a significant knowledge gap in critical care and regional infectious disease surveillance.

Therefore, this study aims to comprehensively characterize the pathogen spectrum and epidemiological features of RTIs in children admitted to PICUs in Wuhan from 2019 to 2024. In addition, we evaluate the performance of multiplex qPCR and other molecular diagnostic tools in real-world critical care settings, focusing on pathogen coverage, positive detection rates, and the capacity to identify coinfections. Our findings will provide valuable evidence to support improved disease management, optimize clinical diagnostic strategies, and guide the development of tailored diagnostic technologies suitable for PICU settings.

## 2. Materials and Methods

### 2.1. Study Design and Population

This retrospective observational study was conducted in the Pediatric Intensive Care Unit (PICU) of Wuhan Children’s Hospital between January 2019 and June 2024; however, due to data loss prior to data acquisition, patient records were not available for certain periods (July 2019–March 2020). The initial PICU database included all pediatric patients admitted during the study period, eligible participants were pediatric patients (aged ≤ 15 years) who had suspected or clinically diagnosed respiratory tract infections (RTIs) and at least one underwent respiratory pathogen testing during hospitalization. Patients without respiratory pathogen testing were excluded. RTI was defined as an infectious disease involving either the upper or lower respiratory tract, caused by viral, bacterial, or other pathogens. Upper respiratory tract infections (URTIs) included the common cold, pharyngitis, tonsillitis, and sinusitis, whereas lower respiratory tract infections (LRTIs) encompassed bronchitis, bronchiolitis, pneumonia, and tracheitis. Diagnosis was based on the presence of typical clinical manifestations—such as fever, cough (productive or non-productive), dyspnea, tachypnea, or chest discomfort—combined with imaging or laboratory findings indicative of pulmonary infection [31,32].

In the present study, admission to the PICU was considered a surrogate marker of disease severity. All included patients required intensive care management for respiratory tract infection, reflecting a critically ill population rather than mild or self-limited RTIs.

### 2.2. Data Collection

Clinical data were retrieved from the hospital’s electronic medical record system. Due to improper data preservation prior to data acquisition, Data from July 2019 to March 2020 were irretrievably lost and could not be included in the analysis, complete patient records were only available for the periods from January to June 2019 and from April 2020 to June 2024. Temporal continuity in graphical and seasonal analyses was preserved by retaining the full time axis and explicitly marking months with unavailable data.

For all eligible patients, demographic information (age and sex), admission date, and relevant clinical information were collected. Patients who underwent respiratory pathogen testing during hospitalization met the inclusion criteria. All pathogen tests were performed as part of routine clinical evaluation during the same hospitalization episode, primarily at or shortly after PICU admission, and were intended to identify pathogens associated with the current episode of respiratory tract infection.

Not all patients underwent all diagnostic modalities. Each included patient received at least one respiratory pathogen test during hospitalization, and some patients underwent multiple testing methods based on clinical judgment, disease severity, and test availability, rather than a predefined hierarchical testing algorithm.

Respiratory pathogen detection was conducted using the following techniques: (1) direct immunofluorescence assay (DFA) for five common respiratory viruses, including respiratory syncytial virus (RSV), human adenovirus (HAdV), influenza A virus (IAV), influenza B virus (IBV), and human parainfluenza viruses (HPIVs); (2) pathogen-specific nucleic acid amplification tests (NAATs) targeting the same five respiratory viruses, as well as *Mycoplasma pneumoniae* (*M. pneumoniae*) and *Chlamydia pneumoniae* (*C. pneumoniae*); (3) conventional microbial culture for bacterial identification, using respiratory specimens including sputum, bronchoalveolar lavage fluid, and throat swabs; and (4) targeted next-generation sequencing (tNGS) for the simultaneous detection of viruses, bacteria, and atypical pathogens, including MP and CP.

For patients tested using multiple diagnostic methods, detection of the same pathogen by different assays was counted only once. Pathogen prevalence was therefore calculated at the patient level, based on the presence or absence of each pathogen per admission, regardless of the number of tests performed.

**Figure 1:**
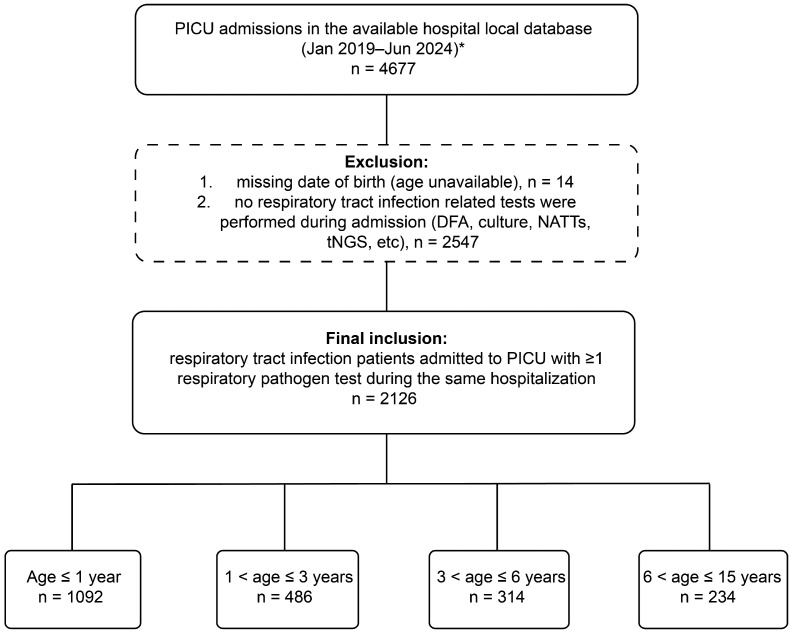
Flowchart of patient selection and age-group stratification. PICU admissions recorded in the available database from January 2019 to June 2024 were screened. * Records from July 2019 to March 2020 were unavailable due to data loss and were excluded from analyses. Eligible participants were RTI patients who underwent at least one respiratory pathogen test during the same hospitalization; patients without respiratory pathogen testing (*n* = 2547) and missing birth date (*n* = 14) were excluded (note: there were 10 patients who met both exclusion criteria). Included patients were stratified into four age groups: age ≤ 1 year, 1 < age ≤ 3 years, 3 < age ≤ 6 years, and 6 < age ≤ 15 years.

Co-infection was defined as the detection of more than one pathogen in the same patient during the same admission, including viral–viral, bacterial–bacterial, or viral–bacterial combinations. Distinction between colonization and true infection was not performed at the microbiological level. All pathogen detections were interpreted in the context of clinical diagnosis, and only patients diagnosed with respiratory tract infection by treating clinicians were included in the analysis. The patient selection process is summarized in Figure 1.

### 2.3. Data Analysis

Four age groups were defined based on immune system development and clinical relevance: baby (≤1 year), infant (1 < age ≤ 3 years), pre-school (3 < age ≤ 6 years), and school-age (6 < age ≤ 15 years). Categorical variables were summarized as counts and percentages. Comparisons of pathogen positivity rates across age groups were performed using the Pearson chi-square test. A two-tailed *p*-value of <0.05 was considered statistically significant. All statistical analyses were performed using R software (version 4.4; R Foundation for Statistical Computing, Vienna, Austria).

### 2.4. Institutional Review Board Statement

The study was conducted in accordance with the ethical standards of the institutional and national research committees and with the Declaration of Helsinki. The protocol was approved by the Ethics Committee of Wuhan Children’s Hospital on 18 June 2025 (Approval No. 2025R086-E01). Due to the retrospective design of the study and the use of de-identified clinical data, the requirement for informed consent was waived.

## 3. Results

### 3.1. Study Population Characteristics

Based on the available PICU admission records, clinical information was obtained for 4677 patients. Exclude 14 patients with missing birth age, then some patients were also excluded because respiratory pathogen testing was not performed at admission. Ultimately, 2126 patients met the inclusion criteria and were included in the final analysis (Figure 1). Among them, 1339 (62.98%) were male and 787 (37.02%) were female, with a male-to-female ratio of approximately 1.70:1. In terms of temporal distribution, 280 patients were enrolled from January to July 2019, 148 from April to December 2020, 408 in 2021, 401 in 2022, 548 in 2023, and 341 from January to June 2024 (Supplementary Table S1). Regarding age distribution, the majority of patients were under 3 years old. Specifically, 1092 patients (51.4%) were ≤1 year old, 486 (22.9%) were 1 < age ≤ 3 years old, 314 (14.8%) were 3 < age ≤ 6 years old, and 234 (11.0%) were 6 < age ≤ 15 years old. In terms of geographic origin, about 94% of patients were from Hubei Province (Figure 1). In Hubei Province, the patients from Wuhan (*n* = 391, 44.33%) are the most numerous. Followed by Huanggang (*n* = 161, 50.16%), Xiaogan (*n* = 115, 51.34%), Suizhou (*n* = 65, 47.45%) and Xianning (*n* = 62, 50.41%) (Supplementary Figure S1).

### 3.2. Age- and Sex-Based Variations in Pathogen Positive Rates of RTIs in PICU

Among the study population, viral infections alone were most frequently detected in the ≤1-year group, with a positivity rate of 28.02%, and showed a marked decline with increasing age: 17.08% in the 1 < age ≤ 3 years group, 13.68% in the 3 < age ≤ 6 years group, and 8.12% in those aged 6 < age ≤ 15 years. In contrast, bacterial infections alone were more prevalent in older children, peaking at 21.34% in the 3 < age ≤ 6 years group and 21.79% in the 6 < age ≤ 15 years group, whereas the ≤1-year group exhibited a lower bacterial detection rate (14.65%). The differences in positivity rates across age groups were statistically significant (χ^2^ test, *p* < 0.05). viral–bacterial co-detection pathogens was more frequently observed in the ≤1-year (11.08%) and 3 < age ≤ 6 years (12.39%) groups. When considering all pathogen categories together, the overall infection burden was highest in the ≤1-year and 3 < age ≤ 6 years groups, with combined positivity rates of 53.75% and 54.70% (Table 1).

In total, 1009 children (47.46%) had at least one respiratory pathogen detected. Among them, 709 cases (33.35%) were mono-infections, while 300 cases (14.11%) involved multiple pathogens (Supplementary Table S1). Multiple viral pathogens were relatively uncommon (5.9% of all viral-positive cases), whereas multiple bacterial pathogens were detected more frequently (17.8% of all bacterial-positive cases). In terms of sex-based differences, males showed a higher overall pathogen positivity rate compared to females (48.17%, 645/1339 vs. 46.12%, 363/787), although the difference was not statistically significant.

### 3.3. Comprehensive Landscape of Pathogen Spectrum in PICU RTI Cases

Among all cases, RSV was the most frequently detected pathogen (18.04%), followed by *S. pneumoniae* (6.95%) (Supplementary Table S1). And the most common mixed detections were *H. influenzae* + RSV (*n* = 32, 3.17%) and *S. pneumoniae* + RSV (*n* = 33, 3.27%).

**Figure 2:**
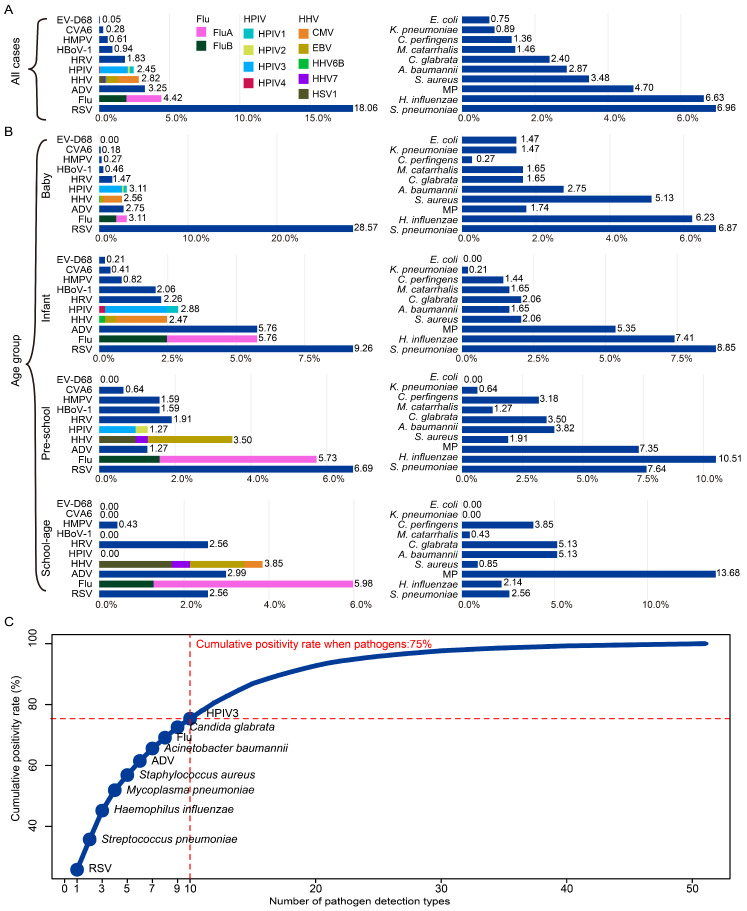
Pathogen landscape of patients with RTIs in PICU of Hubei, 2019–2024. The overall (**A**) and different age group (**B**) composition of top 10 viral and non-viral pathogens detected as positive among 2126 patients admitted to the PICU. The viral pathogen was RSV, Flu, ADV, HHV, HPIV, HRV, HBoV1, HMPV, CVA6, and EV-D68; The non-viral pathogen was *S. pneumoniae*, *H. influenzae*, *M. pneumoniae*, *S. aureus*, *A. baumannii*, *C. glabrata*, *M. catarrhalis*, *C. perfringens*, *K. pneumoniae*, and *E. coli*. The length of the colored bars and the numbers behind indicate the proportion of each pathogen, calculated using the number of positive for the pathogen as the numerator and the number of all PICU respiratory patients as the denominator. For Flu, HPIV, and HHV, the proportion of each subtype is indicated by the color of the bar, for other pathogens without subtype breakdown are displayed using the default color palette and are not intended to convey additional categorical meaning. (**C**) The cumulative positive rate of RTIs-related pathogens detectable in PICU. This curve is plotted based on the cumulative contribution of each pathogen sorted in descending order of positivity rate. The horizontal axis represents the cumulative number of included pathogen species, and the vertical axis represents the corresponding cumulative positive detection rate. Each point represents a newly added pathogen.

Focusing on viral pathogens, the top 10 viruses ranked by overall positivity were: RSV (18.06%), Flu (4.42%), ADV (3.25%), human herpesviruses (HHV, 2.82%), HPIV (2.45%), HRV (1.83%), human bocavirus (HBoV1, 0.94%), HMPV (0.61%), coxsackievirus A6 (CVA6, 0.28%), and enterovirus D68 (EV-D68, 0.05%) (Figure 2A). Subtyping revealed that Flu A (2.49%) was slightly more common than Flu B (1.93%), and among HPIVs, HPIV3 (1.98%) dominated over HPIV1 (0.28%), HPIV2 (0.14%), and HPIV4 (0.05%). Cytomegalovirus (CMV, 1.51%) was the leading genotype within HHVs, followed by EVB (0.08%), HSV1 (0.33%), HHV6B (0.09%), and HHV7 (0.09%). Further age-specific analysis showed that RSV ranked first across 3 age groups (≤6 years), especially among babies (28.57%), then decreased by aging. In school-age children, Flu ranked first with positive rate of 5.98. Notably, Flu and HHV prevalence increased with age, peaking in school-aged children (Figure 2B). The remaining virus rankings varied depending on the age group.

The top 10 non-viral pathogens showed a different distribution. *S. pneumoniae* (6.96%) remained the most common in total, followed by *H. influenzae* (6.63%), MP (4.70%), *S. aureus* (3.48%), *Acinetobacter baumannii* (*A. baumannii*, 2.87%), and *Candida glabrata* (*C. glabrata*, 2.40%). Less frequent pathogens included *Moraxella catarrhalis* (*M. catarrhalis*, 1.46%), *Clostridium perfringens* (*C. perfringens*, 1.36%), *Klebsiella pneumoniae* (*K. pneumoniae*, 0.89%), and *Escherichia coli* (*E. coli*, 0.75%) (Figure 2A). Age-stratified analysis revealed unique trends: *S. pneumoniae* and *H. influenzae* were most prevalent pathogens in children under age 6, while in school-aged children, MP became dominant, accompanied by increased detection of *A. baumannii*, *C. glabrata*, and *C. perfringens*. It is worth noting that the positive rate of MP increases with age. Furthermore, E. coli was only detected in the baby group (Figure 2B).

To evaluate the diagnostic value and screening efficiency of pathogen detection in the PICU setting under current detection methods, cumulative positivity rate analysis was conducted. Results revealed that the top 10 most frequently detected respiratory pathogens—including RSV, *S. pneumoniae*, *H. influenzae*, MP, *S. aureus*, ADV, *A. baumannii*, IAV, *C. glabrata*, and HPIV3—accounted for approximately 75% of all positive detections in children with RTIs admitted to the PICU (Figure 2C).

### 3.4. Patterns of Age-Specific Positive Number and Positive Rate of RTIs Related Pathogens in PICU

**Figure 3:**
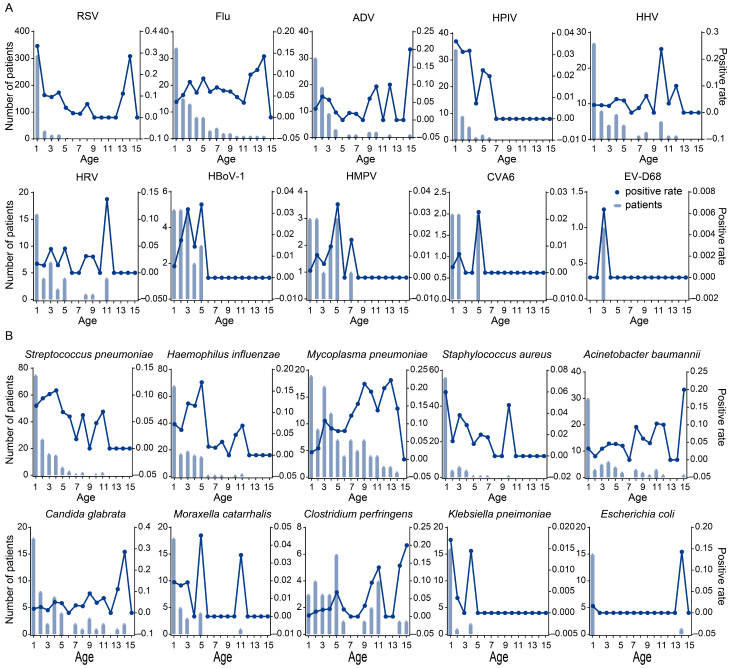
The number and rate of pathogen-positive cases in PICU with RTIs that change with age. (**A**) Top 10 viral pathogens. (**B**) Top 10 bacterial pathogens. The Bars indicate the number of pathogen-positive patients at each single year of age. Points and connecting lines represent the positivity rate, defined as the proportion of patients testing positive for a given pathogen among all PICU patients within the same age year. Age was treated as completed years; for example, age 1 represents children aged > 0 and ≤1 year, and subsequent ages were defined accordingly. Age is presented at single-year resolution for descriptive visualization of age-related patterns.

Among the 2126 PICU patients tested for viral pathogens, 650 (30.58%) were confirmed to be virus-positive based on the detection of at least one viral target. The distribution of viral infections exhibited significant age-related differences. RSV exhibited a sharply elevated positive number and rate in early infancy under age 1, followed by a steady decline with age, and a slight increase in positive rate around age 13–14. The number of positive cases of Flu and ADV shows a downward trend with increasing age, and the positive rate fluctuates without certain pattern. HPIV were primarily detected in children under 5, and positivity declined to near zero in older children. HHV and HRV showed higher numbers of positive cases in younger children, while sporadic higher positivity rates were observed in certain older age groups. HBoV-1 was mainly detected under age 5, with relatively stable positivity. HMPV appeared sporadically, with small clusters between ages under 5 and around 7–8. CVA6 and EV-D68 were rarely detected, with isolated cases showing very low positivity rates (Figure 3A).

While, 569 PICU patients detected at least one non-viral pathogen. The distribution also varied significantly across age groups. *S. pneumoniae* and *H. influenzae* were most common in children under 5, both in case numbers and positivity rates. MP cases were observed across most ages, except at age 15, with relatively higher positivity rates noted at ages 9 and 13. *S. aureus*, *A. baumannii*, *C. perfringens*, *M. catarrhalis*, *K. pneumoniae*, and *E. coli* were primarily detected in children under 1. In contrast, and *C. glabrata* displayed fluctuating detection across all ages (Figure 3B).

### 3.5. Temporal Dynamics and Seasonal Trends of RTIs Related Pathogens in PICU

**Figure 4:**
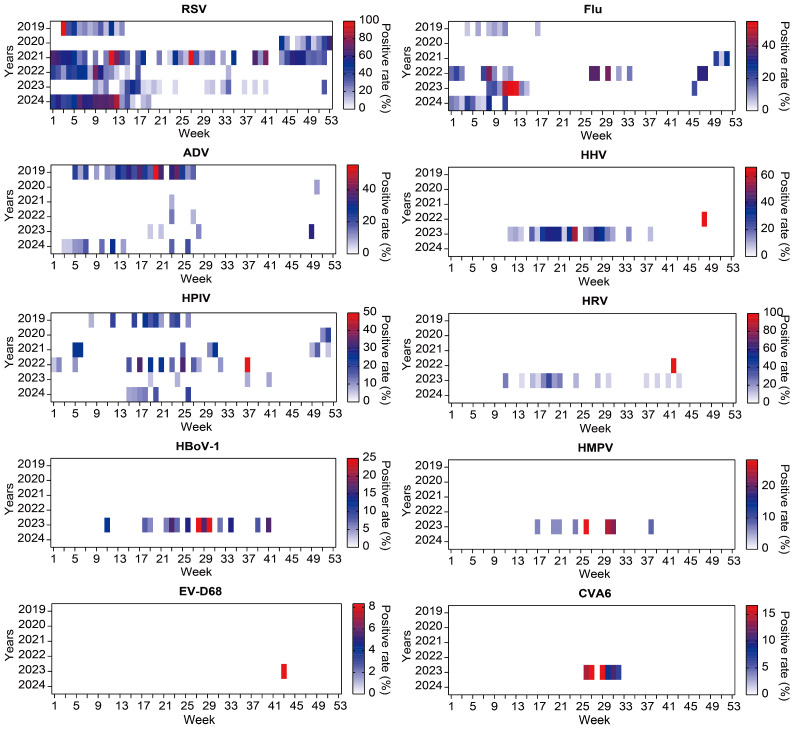
Seasonal patterns of the top 10 viral respiratory pathogens among PICU patients with RTIs, 2019–2024. Heatmaps display the weekly positivity rate (%) of each viral pathogen across calendar weeks and study years. The positivity rate was calculated on a weekly basis, defined as the number of patients testing positive for a given pathogen divided by the total number of PICU patients tested for respiratory pathogens in the same week. Color intensity indicates the magnitude of the weekly positivity rate. Data from July 2019 to March 2020 were unavailable due to data loss prior to acquisition and were not included in the analyses. The full time axis was retained for visualization to preserve temporal continuity, with the missing period explicitly indicated rather than imputed as zero.

Viral pathogens exhibited distinct seasonal and temporal variations, with several viruses showing epidemic surges during specific years and seasons. Across the study period, nearly all respiratory viruses—except HPIV2—were detected in 2023, which also marked the most active year for viral pathogen detection. RSV showed winter and spring prevalence in year 2019, 2020, 2022 and 2024, while a year-round prevalence in 2021 and a weakened summer and autumn epidemic in 2023. Flu displayed classic seasonal characteristics during winter and spring, with disappearance in 2020–2021 and abnormal rising in summer 2022. ADV were highly prevalent in the spring and summer of 2019, and had a low prevalence during the same period in 2024, while they were sporadic in other years. HPIV, particularly HPIV3, was predominantly detected during spring and summer, especially in 2019, 2022 and 2024. HPIV1, HPIV4, HMPV, EV-D68, EVB, HSV1, CMV, and HHV subtypes, were sporadically detected—mostly in 2022 and 2023 (Figure 4, Supplementary Figure S2).

**Figure 5:**
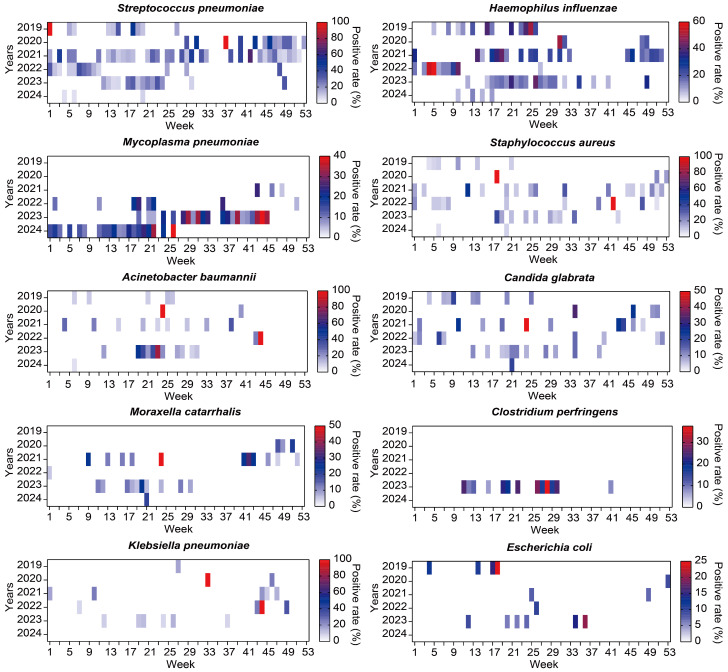
Seasonal patterns of the top 10 non-viral (bacterial and fungal) respiratory pathogens among PICU patients with RTIs, 2019–2024. Heatmaps display the weekly positivity rate (%) of each pathogen across calendar weeks and study years. The positivity rate was calculated on a weekly basis, using the number of positive detections divided by the total number of tested PICU patients per week. Color intensity represents the weekly positivity rate. Data from July 2019 to March 2020 were unavailable due to data loss prior to acquisition and were not included in the analyses. The full time axis was retained for visualization to preserve temporal continuity, with the missing period explicitly indicated rather than imputed as zero.

Non-viral pathogens do not have obvious seasonal epidemic patterns, and their occurrence varies more significantly from year to year. Major bacteria such as *S. pneumoniae*, *H. influenzae* and *S. aureus* were all detected (with inevitable data deficiency in 2019 and 2020), but there was no obvious seasonal pattern, and the prevalence was low in the second half of 2022. The positive rate of MP has sharply increased since the second half of 2021. *A. baumannii* and *C. glabrata* mainly experienced sporadic cases in 2019, 2021 and 2023 during the non-winter period. *M. catarrhalis*, *C. perfringens*, *K. pneumoniae*, and *E. coli* were all highly prevalent in the summer of 2023, but the high-prevalence seasons varied in other years (Figure 5). Several opportunistic or rare pathogens, including *Bordetella pertussis*, *Pseudomonas putida*, *Tropheryma whipplei*, *Pneumocystis jirovecii*, and *Aspergillus fumigatus*, were only sporadically detected in 2023, with most peaks occurring in spring and summer, indicating episodic or outbreak-like features. Conversely, certain pathogens such as *Candida albicans*, *Enterococcus faecalis*, *Aspergillus* spp., and *Escherichia hermannii* were observed only prior to the pandemic (e.g., in 2019), and were absent during the epidemic period. Others, like *Corynebacterium diphtheriae* and *Serratia marcescens*, appeared briefly in 2020, while *Candida tropicalis*, once prevalent in spring and winter, disappeared after 2021 (Supplementary Figure S3).

## 4. Discussion

Current research on the etiological spectrum of respiratory infections in PICU populations in China remains insufficient, with most studies focusing on outpatients or general inpatients [20,21,24]. This knowledge gap underscores the necessity of gaining a deeper understanding of pathogen distributions in critically ill children. To address this, the present study analyzed respiratory samples collected from PICU patients with respiratory infections at Wuhan Children’s Hospital between 2019 and 2024, systematically examining the detection patterns, age distributions, and seasonal trends of viruses, bacteria, and Mycoplasma. The findings not only fill a critical gap in pathogen surveillance for PICU populations in central China but also reveal distinct epidemiological variations across age groups and temporal dimensions, demonstrating pathogen characteristics specific to severe cases. Moreover, these results provide valuable evidence to inform the future development and application of efficient multi-target molecular diagnostic technologies [23,33].

Among all detected pathogens, RSV, S. pneumoniae, and H. influenzae were the most frequently identified, followed by MP, ADV, and others with relatively lower detection rates. Furthermore, cumulative positivity rate analysis based on current clinical diagnostic results showed that the top ten most prevalent pathogens accounted for over 75% of positive cases, indicating that most respiratory infections in the PICU can be effectively captured by targeting a limited set of pathogens. On this basis, developing multiplex detection methods [33,34,35,36,37] tailored to these high-frequency pathogens and specific to the PICU setting would not only reduce resource consumption and healthcare costs but also reduce unnecessary antibiotic exposure and resource utilization in pediatric settings [38].

This study identified clear age-dependent distribution patterns of major respiratory pathogens among PICU patients. RSV predominated in baby, IFV and ADV were most frequent in school-aged children, whereas MP was more common in school-aged children, and *S. pneumoniae* and *H. influenzae* were largely concentrated in ≤6 years. From a clinical perspective, these age-specific patterns have important implications for PICU practice. Baby represent a particularly vulnerable population for severe RSV [39] and invasive bacterial infections, underscoring the need for heightened surveillance, early diagnostic testing, and timely supportive management in this age group. It should be noted that, in this study, pathogen categories were defined based on routine clinical diagnostic results rather than a standardized testing algorithm. Testing intensity may have varied according to age and disease severity, particularly in critically ill patients, potentially increasing the likelihood of detecting co-infections in these groups. Therefore, the observed higher proportion of viral–bacterial co-detections in younger children may partly reflect diagnostic practice patterns rather than true biological differences alone. In contrast, the predominance of IFV, ADV, and MP [40] in older children [41] suggests that pathogens often considered “common” in outpatient settings may be associated with disproportionate severity once critical illness develops, warranting early recognition and aggressive monitoring in the PICU. From a preventive and public health standpoint, these findings support age-stratified diagnostic and management strategies, including prioritization of targeted testing panels, tailored infection prevention measures, and optimized allocation of PICU resources during high-risk periods. Similar age-related vulnerability patterns have been reported in both community-based and hospital-based pediatric studies, reinforcing the relevance of age as a key determinant of pathogen burden and disease severity in critically ill children.

From a global public health perspective, seasonal distribution analyses revealed broadly consistent epidemic patterns for major viral pathogens, including winter–spring predominance of RSV and periodic surges of influenza viruses. However, temporal heterogeneity was observed across the study period, particularly in relation to the COVID-19 pandemic. Although formal pre-/post-pandemic statistical comparisons or trend testing were not performed because of a structural data gap in 2019–2020 and evolving diagnostic practices (including changes in testing panels/platforms and pathogen coverage) that limited cross-period comparability, several descriptive patterns merit discussion. In the pre-pandemic year (2019), RSV and influenza exhibited typical winter–spring seasonality, whereas ADV and HPIV (especially HPIV3) were more frequently detected during spring and summer. During the pandemic period (2020–2021), circulation of several respiratory viruses—particularly influenza—appeared attenuated or atypical, consistent with widely reported effects of non-pharmaceutical interventions on viral transmission [42,43]. Within the limits of our available data, viral activity during this period was reduced or irregular compared with other years. In the post-pandemic period (2022–2024), broader viral activity re-emerged, with 2023 representing the most active year for viral detection in our cohort, during which nearly all monitored viruses were identified. Notably, increased detection of less commonly reported viruses, including HHV, EV-D68, and CVA6, was observed [44]. Similar post-pandemic resurgence and diversification of respiratory viruses have been reported in multiple regions, including atypical timing and increased intensity of RSV epidemics following the relaxation of mitigation measures [45]. Taken together, these observations suggest that large-scale public health interventions, such as lockdowns and other non-pharmaceutical measures implemented during the COVID-19 pandemic, may substantially influence respiratory pathogen circulation patterns. However, given the structural data gap and time-varying diagnostic practices across the study period, these observations should be interpreted cautiously.

However, this study has several limitations. First, as a single-center retrospective study, its regional applicability requires validation in multicenter settings. Second, despite employing qPCR and NGS methodologies, detection rates for some rare or emerging pathogens remained low, possibly underestimating their presence. Third, temporal inference was constrained by a structural data gap (June 2019–June 2020) and by evolving diagnostic strategies over time (including changes in assay panels/platforms and pathogen coverage), which limited cross-period comparability; accordingly, temporal and seasonal patterns were presented descriptively without formal trend testing or pre-/post-pandemic statistical comparisons. Lastly, clinical outcomes (e.g., severity scores, length of stay, mortality) were not analyzed in relation to pathogen composition; future studies should incorporate such data to enable stratified precision care.

In conclusion, this study elucidates the pathogen spectrum of severe pediatric respiratory infections in the PICU in Hubei Province and identifies age- and season-specific epidemiological characteristics. These findings have practical implications for optimizing diagnostic and empirical treatment strategies. The establishment of targeted detection panels for high-frequency pathogens (e.g., RSV, S. pneumoniae, ADV) could improve diagnostic yield and cost-effectiveness. Moreover, we recommend integrating an “age–season–high-risk pathogen” model into standard PICU diagnostic workflows to facilitate the transition from empirical management to standardized, data-driven precision care in pediatric infectious disease control. These findings also provide evidence to support adaptive surveillance strategies and dynamic updating of diagnostic panels in response to changing epidemiological contexts, including post-pandemic pathogen resurgence and emerging respiratory threats.

## 5. Conclusions

In summary, this study characterizes the pathogen spectrum of severe pediatric respiratory infections in a PICU setting and highlights marked heterogeneity across age groups and pathogen categories that is specific to critically ill children. The consistently high burden observed in younger age groups underscores the importance of age-informed risk stratification when interpreting respiratory pathogen findings in the PICU. Rather than relying on broad, undifferentiated testing strategies, our results support a more targeted diagnostic approach focused on a limited number of high-frequency pathogens that account for the majority of detected infections. Such an approach may improve diagnostic efficiency and clinical relevance while reducing unnecessary testing in resource-intensive critical care settings. Collectively, these findings provide practical epidemiological evidence to inform PICU-oriented pathogen surveillance and the rational design of multiplex diagnostic panels, thereby supporting a transition toward more standardized and data-driven management of severe pediatric respiratory infections.

## Figures and Tables

**Table 1:** Positive rates of viral and bacterial pathogens in patients with RTIs in PICU in Wuhan, 2019–2024.

	Age Group				*p* Value	Detection Rate (N = 2126)		
	Age ≤ 1 Year	1 < Age ≤ 3 Years	3 < Age ≤ 6 Years	6 < Age ≤ 15 Years		All	Single Detection	Multiple Detection
Total patients	1092	486	314	234	-	-	-	-
Viral-only positive	306 (28.02)	83 (17.08)	32 (13.68)	19 (8.12)	<0.001	440 (20.70)	414 (19.47)	26 (1.22)
Bacterial-only positive	160 (14.65)	81 (16.67)	67 (21.34)	51 (21.79)	0.006	359 (16.89)	295 (13.88)	64 (3.01)
viral–bacterial co-detection	121 (11.08)	46 (9.47)	29 (12.39)	14 (5.98)	0.111	210 (9.88)	-	-
All	587 (53.75)	210 (43.21)	128 (54.70)	84 (35.90)	<0.001	1009 (47.46)	709 (33.35)	300 (14.11)

Note: “-” indicates *not applicable*.

## Data Availability

The data presented in this study are available within the article and its Supplementary Materials. Additional information or detailed datasets can be provided by the corresponding authors upon reasonable request.

## Supplementary Materials

The following supporting information can be downloaded at: https://www.mdpi.com/article/10.3390/pathogens15020219/s1, **Supplementary Table S1**: Positive rates of related pathogens in patients with RTIs in PICU in Wuhan, 2019–2024; **Supplementary Figure S1**: Geographic composition and distribution of enrolled cases. The pie chart shows the composition of the source provinces included in this study. The map shows the distribution of cases within the included Hubei Province by city; **Supplementary Figure S2**: Seasonal Analysis of Respiratory Infection Pathogens in the PICU. Viral pathogens of respiratory infections detected in PICUs, 2019–2024; **Supplementary Figure S3**: Seasonal Analysis of Respiratory Infection Pathogens in the PICU. Bacterial pathogens of respiratory infections detected in PICUs, 2019–2024.
