# Supplementary material for: Respiratory Infection-Related Pathogens in the Pediatric Intensive Care Unit During 2019–2024 in Hubei, China
Source: Pathogens. 2026 Feb 14;15(2):219. doi: 10.3390/pathogens15020219 (PMC12942933; doi:10.3390/pathogens15020219)
Supplement: Supplementary file 1 [file pathogens-15-00219-s001.zip › Supplementary Table S1.pdf]

Supplementary Table S1

Positive rates of related pathogens in patients with RTIs in PICU in Wuhan, 2019-2024.

	2019(n=280)	2020(n=148)	2021(n=408)	2022(n=401)	2023(n=548)	2024(n=341)	Total(n=2126)
**Sex**							
Male(n=1339)	92(4.33)	34(1.60)	153(7.20)	100(4.70)	151(7.10)	115(5.41)	645(30.33)
**Age**							
≤1 year old (n=1092)	88(4.14)	35(1.65)	151(7.10)	87(4.09)	104(4.89)	122(5.74)	587(27.61)
1 < age ≤ 3 years old (n=486)	41(1.93)	15(0.71)	43(2.02)	34(1.60)	48(2.26)	29(1.36)	210(9.88)
3 < age ≤ 6 years old (n=314)	9(0.42)	6(0.28)	26(1.22)	19(0.89)	51(2.40)	17(0.80)	128(6.02)
6 < age ≤ 15 years old (n=234)	8(0.38)	2(0.09)	8(0.38)	11(0.52)	39(1.83)	16(0.75)	84(3.95)
**Detection rate summary**							
Detection rate	146(44.19)	58(39.19)	228(55.88)	152(37.91)	240(43.80)	185(54.25)	1009(47.46)
Single detection	123(43.93)	47(31.76)	161(39.46)	108(26.93)	114(20.80)	156(45.75)	709(33.35)
Multiple detection	23(8.21)	11(7.43)	67(16.42)	44(10.97)	126(22.99)	29(8.50)	300(14.11)
Virus	93(33.	23(15.	130(31.	108(26	147(26	149(43	650(30.5

detection rate	21)	54)	86)	.93)	.82)	.70)	7)
Single virus detection	90(32.14)	23(15.54)	129(31.62)	102(25.44)	99(18.07)	134(39.30)	577(27.14)
Multiple virus detection	3(1.07)	0(0.00)	1(0.25)	6(1.50)	48(8.76)	15(4.40)	73(3.43)
Bacteria detection rate	67(23.93)	40(27.03)	156(38.24)	80(19.95)	174(31.75)	52(15.25)	569(26.76)
Single bacteria detection	57(20.36)	33(22.30)	139(34.07)	67(16.71)	86(15.69)	50(14.66)	432(20.32)
Multiple bacteria detection	10(3.57)	7(4.73)	17(4.17)	13(3.24)	88(16.06)	2(0.59)	137(6.44)
**Single pathogen positive rate**							
HPIV1	0(0.00)	0(0.00)	1(0.24)	4(1.00)	1(0.18)	0(0.00)	6(0.28)
HPIV2	1(0.36)	0(0.00)	0(0.00)	2(0.50)	0(0.00)	0(0.00)	3(0.14)
HPIV3	11(3.93)	2(1.35)	9(2.20)	11(2.74)	2(0.36)	7(2.05)	42(1.97)
HPIV4	0(0.00)	0(0.00)	0(0.00)	0(0.00)	1(0.18)	0(0.00)	1(0.05)
FluA	6(2.14)	0(0.00)	0(0.00)	20(4.98)	22(4.01)	5(1.46)	53(2.49)
FluB	1(0.36)	0(0.00)	8(1.96)	16(3.98)	1(0.18)	15(4.39)	41(1.93)
ADV	41(14.64)	1(0.68)	1(0.24)	2(0.50)	5(0.91)	19(5.56)	69(3.24)

RSV	36(12.86)	19(12.84)	113(27.63)	56(13.93)	42(7.66)	118(34.50)	384(18.04)
HMPV	0(0.00)	0(0.00)	0(0.00)	0(0.00)	13(2.37)	0(0.00)	13(0.61)
HHV6B	0(0.00)	0(0.00)	0(0.00)	0(0.00)	2(0.36)	0(0.00)	2(0.09)
HHV7	0(0.00)	0(0.00)	0(0.00)	1(0.25)	1(0.18)	0(0.00)	2(0.09)
HSV1	0(0.00)	0(0.00)	0(0.00)	1(0.25)	6(1.09)	0(0.00)	7(0.33)
CMV	0(0.00)	0(0.00)	0(0.00)	0(0.00)	32(5.84)	0(0.00)	32(1.50)
CVA6	0(0.00)	0(0.00)	0(0.00)	0(0.00)	6(1.09)	0(0.00)	6(0.28)
HBoV-1	0(0.00)	0(0.00)	0(0.00)	0(0.00)	20(3.65)	0(0.00)	20(0.94)
HRV	0(0.00)	0(0.00)	0(0.00)	1(0.25)	38(6.93)	0(0.00)	39(1.83)
EV-D68	0(0.00)	0(0.00)	0(0.00)	0(0.00)	1(0.18)	0(0.00)	1(0.05)
EBV	0(0.00)	0(0.00)	0(0.00)	0(0.00)	17(3.10)	0(0.00)	17(0.80)
Chlamydia pneumoniae	0(0.00)	0(0.00)	1(0.24)	1(0.25)	0(0.00)	1(0.29)	3(0.14)
Escherichia coli	6(2.14)	1(0.68)	2(0.49)	1(0.25)	6(1.09)	0(0.00)	16(0.75)
Enterobacter aerogenes	0(0.00)	0(0.00)	0(0.00)	0(0.00)	1(0.18)	0(0.00)	1(0.05)
Candida	1(0.36)	0(0.00)	0(0.00)	0(0.00	0(0.00)	0(0.00)	1(0.05)

albicans				)			
Corynebacterium diphtheriae	0(0.00)	1(0.68)	0(0.00)	0(0.00)	0(0.00)	0(0.00)	1(0.05)
Candida tropicalis	3(1.07)	1(0.68)	1(0.24)	0(0.00)	0(0.00)	0(0.00)	5(0.23)
Serratia marcescens	0(0.00)	1(0.68)	0(0.00)	0(0.00)	0(0.00)	0(0.00)	1(0.05)
Aspergillus spp	2(0.71)	0(0.00)	0(0.00)	0(0.00)	0(0.00)	0(0.00)	2(0.09)
Enterococcus faecalis	2(0.71)	0(0.00)	0(0.00)	0(0.00)	0(0.00)	0(0.00)	2(0.09)
Escherichia hermannii	2(0.71)	0(0.00)	0(0.00)	0(0.00)	0(0.00)	0(0.00)	2(0.09)
Candida parapsilosis	1(0.36)	0(0.00)	1(0.24)	0(0.00)	6(1.09)	0(0.00)	8(0.38)
Clostridium perfringens	0(0.00)	0(0.00)	0(0.00)	0(0.00)	29(5.29)	0(0.00)	29(1.36)
Aspergillus fumigatus	0(0.00)	0(0.00)	0(0.00)	0(0.00)	1(0.18)	0(0.00)	1(0.05)
Aspergillus niger	0(0.00)	0(0.00)	0(0.00)	1(0.25)	0(0.00)	0(0.00)	1(0.05)
Streptococcus constellatus	0(0.00)	0(0.00)	0(0.00)	0(0.00)	1(0.18)	0(0.00)	1(0.05)
Streptococcus intermedius	0(0.00)	0(0.00)	0(0.00)	0(0.00)	2(0.36)	0(0.00)	2(0.09)
Bordetella pertussis	0(0.00)	0(0.00)	0(0.00)	0(0.00)	5(0.91)	0(0.00)	5(0.23)
Ureaplasma	0(0.00)	0(0.00)	0(0.00)	0(0.00	1(0.18)	0(0.00)	1(0.05)

urealyticum				)			
Pseudomonas putida	0(0.00)	0(0.00)	0(0.00)	0(0.00)	5(0.91)	0(0.00)	5(0.23)
Tropheryma whipplei	0(0.00)	0(0.00)	0(0.00)	0(0.00)	3(0.55)	0(0.00)	3(0.14)
Pneumocystis jirovecii	0(0.00)	0(0.00)	1(0.25)	0(0.00)	7(1.28)	0(0.00)	8(0.38)
Chlamydia trachomatis	0(0.00)	0(0.00)	1(0.25)	0(0.00)	0(0.00)	0(0.00)	1(0.05)
Mycoplasma pneumoniae	0(0.00)	0(0.00)	3(0.73)	9(2.24)	51(9.31)	37(10.85)	100(4.70)
Streptococcus pneumoniae	12(4.29)	21(14.19)	53(12.96)	23(5.72)	35(6.39)	4(1.17)	148(6.95)
Candida glabrata	9(3.21)	4(2.70)	11(2.69)	9(2.24)	16(2.92)	2(0.58)	51(2.40)
Haemophilus influenzae	24(8.57)	5(3.38)	47(11.49)	26(6.47)	35(6.39)	4(1.17)	141(6.62)
Klebsiella pneumoniae	1(0.36)	2(1.35)	5(1.22)	3(0.75)	8(1.46)	0(0.00)	19(0.89)
Staphylococcus aureus	8(2.86)	5(3.38)	22(5.38)	16(3.99)	20(3.65)	3(0.88)	74(3.48)
Streptococcus pyogenes	0(0.00)	0(0.00)	1(0.24)	0(0.00)	9(1.64)	0(0.00)	10(0.47)
Acinetobacter baumannii	5(1.79)	2(1.35)	10(2.44)	1(0.25)	42(7.66)	2(0.59)	62(2.91)
Pseudomonas aeruginosa	1(0.36)	0(0.00)	3(0.73)	3(0.75)	7(1.28)	1(0.29)	15(0.70)
Haemophilus	0(0.00)	0(0.00)	2(0.49)	1(0.25	0(0.00)	0(0.00)	3(0.14)

haemolyticus				)			
Moraxella catarrhalis	0(0.00)	4(2.70)	11(2.70)	1(0.25)	14(2.55)	1(0.29)	31(1.46)
