# Supplementary figures and images for: Respiratory Infection-Related Pathogens in the Pediatric Intensive Care Unit During 2019–2024 in Hubei, China
Source: Pathogens. 2026 Feb 14;15(2):219. doi: 10.3390/pathogens15020219 (PMC12942933; doi:10.3390/pathogens15020219)

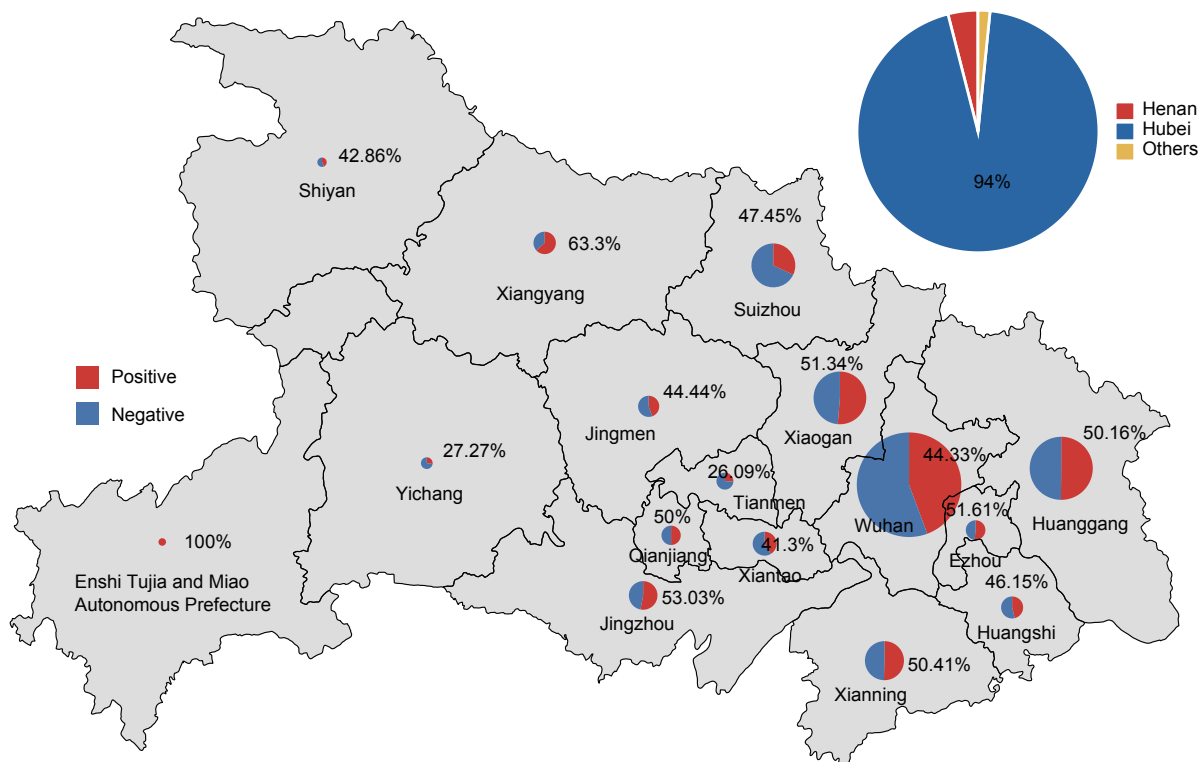

Supplement: Supplementary file 1 [file pathogens-15-00219-s001.zip › Supplementary Figure. S1.pdf]

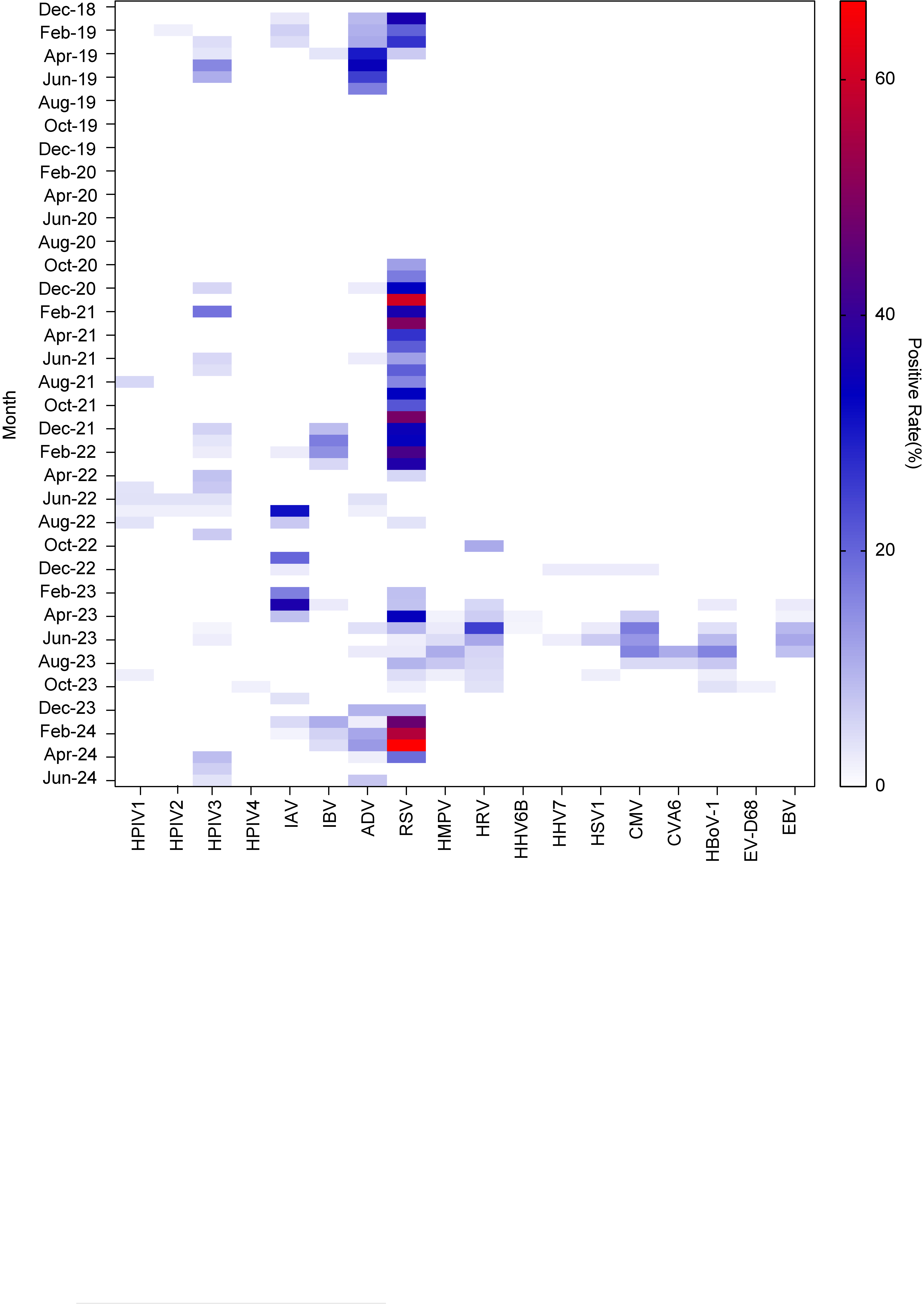

Supplement: Supplementary file 1 [file pathogens-15-00219-s001.zip › Supplementary Figure. S2.jpg]

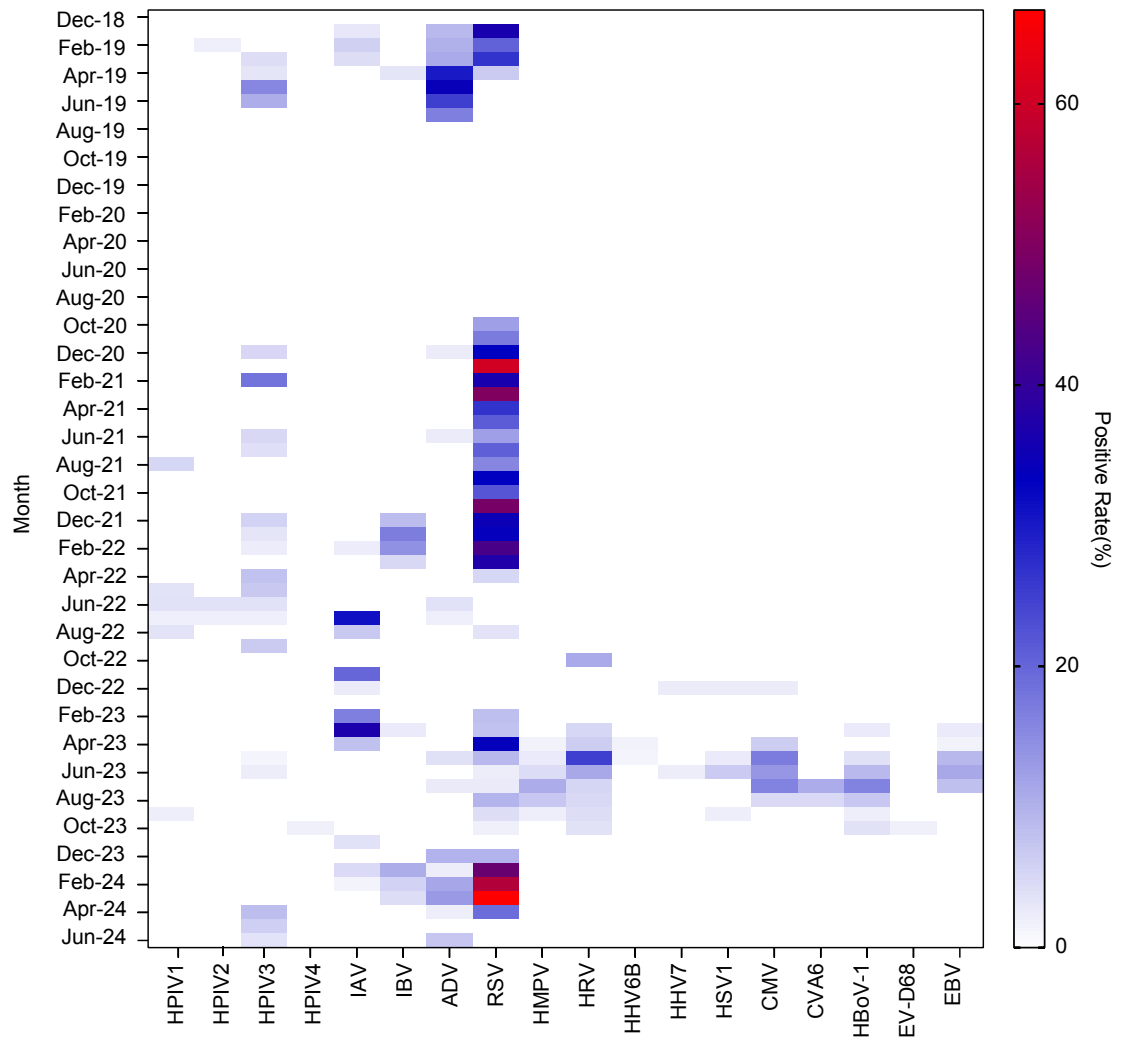

Supplement: Supplementary file 1 [file pathogens-15-00219-s001.zip › Supplementary Figure. S2.pdf]

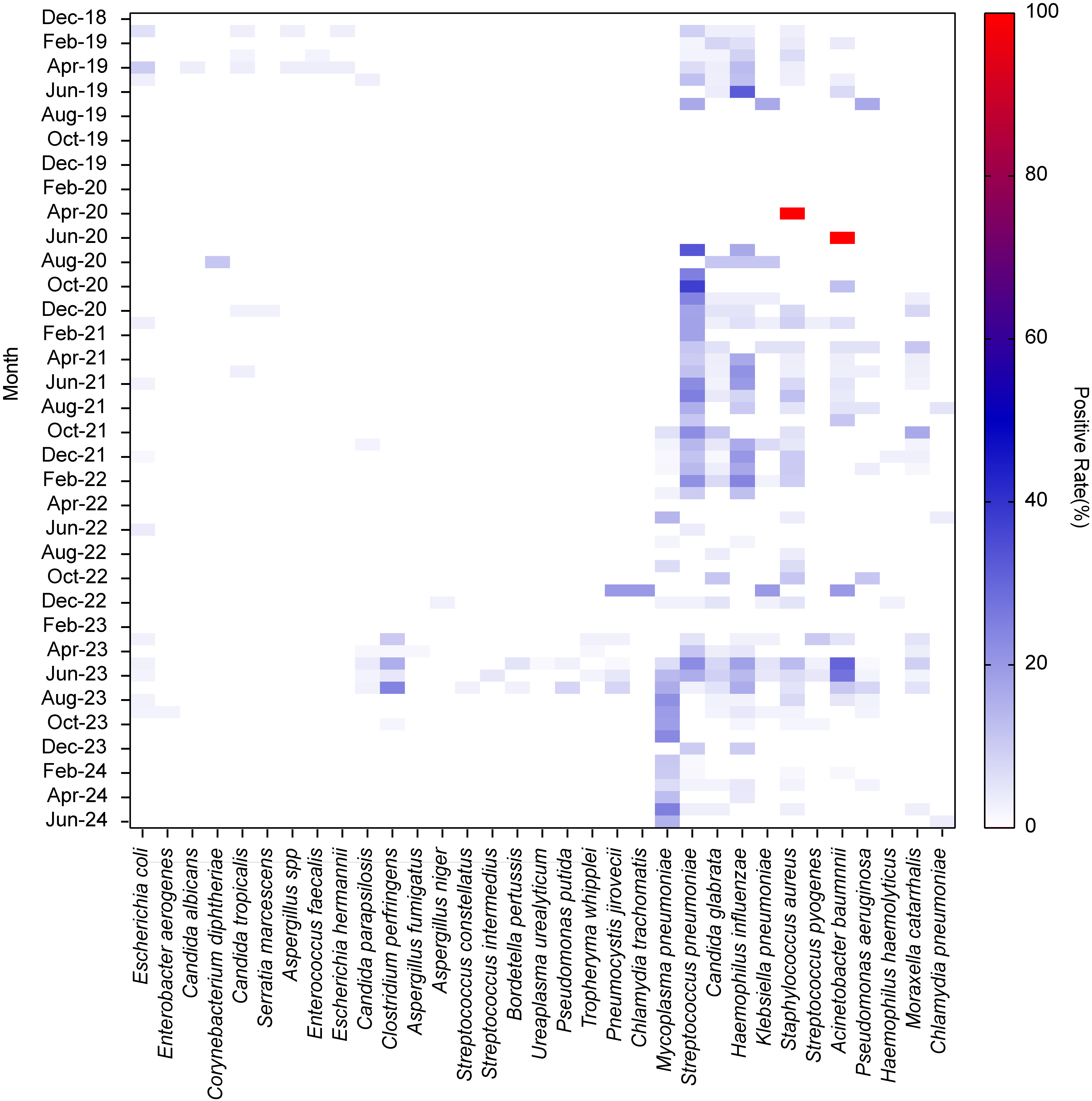

Supplement: Supplementary file 1 [file pathogens-15-00219-s001.zip › Supplementary Figure. S3.jpg]

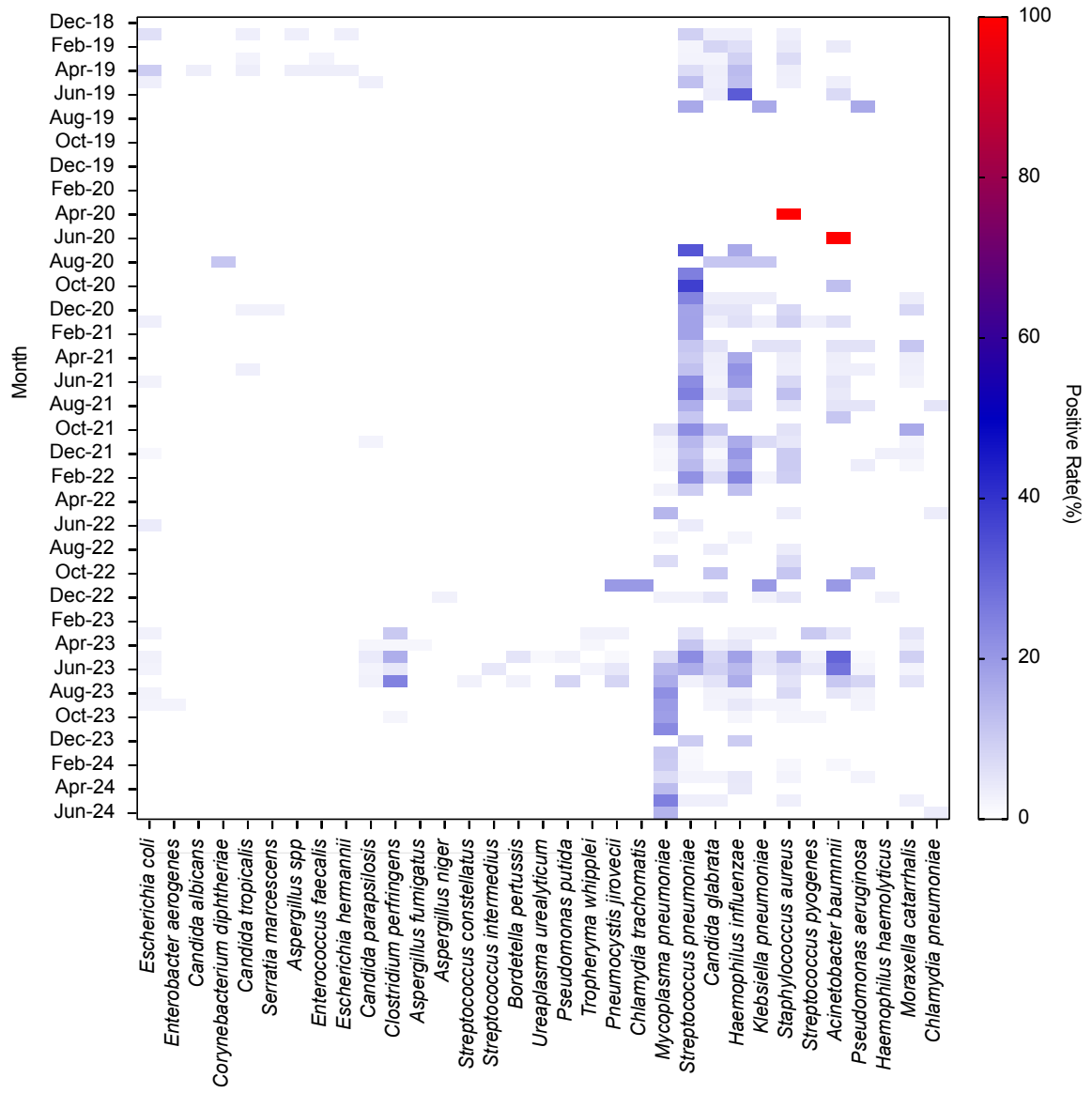

Supplement: Supplementary file 1 [file pathogens-15-00219-s001.zip › Supplementary Figure. S3.pdf]
